# Prediction of chronic kidney disease progression using recurrent neural network and electronic health records

**DOI:** 10.1038/s41598-023-49271-2

**Published:** 2023-12-13

**Authors:** Yitan Zhu, Dehua Bi, Milda Saunders, Yuan Ji

**Affiliations:** 1 Computing, Environment and Life Sciences, Argonne National Laboratory, 9700 S Cass Ave, Lemont, IL 60439 USA; 2 Department of Public Health Sciences, The University of Chicago, 5841 South Maryland Ave, MC 2000, Chicago, IL 60637 USA; 3 Department of Medicine, The University of Chicago, 5841 South Maryland Ave, MC 2007, Chicago, IL 60637 USA

**Keywords:** Computational models, Machine learning, Data processing, Chronic kidney disease, Predictive markers

## Abstract

Chronic kidney disease (CKD) is a progressive loss in kidney function. Early detection of patients who will progress to late-stage CKD is of paramount importance for patient care. To address this, we develop a pipeline to process longitudinal electronic heath records (EHRs) and construct recurrent neural network (RNN) models to predict CKD progression from stages II/III to stages IV/V. The RNN model generates predictions based on time-series records of patients, including repeated lab tests and other clinical variables. Our investigation reveals that using a single variable, the recorded estimated glomerular filtration rate (eGFR) over time, the RNN model achieves an average area under the receiver operating characteristic curve (AUROC) of 0.957 for predicting future CKD progression. When additional clinical variables, such as demographics, vital information, lab test results, and health behaviors, are incorporated, the average AUROC increases to 0.967. In both scenarios, the standard deviation of the AUROC across cross-validation trials is less than 0.01, indicating a stable and high prediction accuracy. Our analysis results demonstrate the proposed RNN model outperforms existing standard approaches, including static and dynamic Cox proportional hazards models, random forest, and LightGBM. The utilization of the RNN model and the time-series data of previous eGFR measurements underscores its potential as a straightforward and effective tool for assessing the clinical risk of CKD patients concerning their disease progression.

## Introduction

Chronic kidney disease (CKD) is a progressive loss in kidney function over a period of months or years. It is differentiated from acute kidney disease in that the reduction in kidney function must be present for over three months. CKD is an internationally recognized public health problem affecting 5–10% of the world population^1,2^. The disease is graded into five stages based on the estimated glomerular filtration rate (eGFR) value. The eGFR is a measure of the kidney function, derived by a math formula using a person’s age, sex and serum creatinine. According to eGFR values, CKD can be categorized as: stage I (eGFR ≥ 90), stage II (89 ≥ eGFR ≥ 60), stage III (59 ≥ eGFR ≥ 30), stage IV (29 ≥ eGFR ≥ 15), and stage V (eGFR < 15). Stages I through III are early stages, and stages IV and V are late and end stages, respectively. Treatment strategies for CKD patients are tailored according to their CKD stages. If CKD is accurately identified at an early stage, medicines and lifestyle changes may help slow its progress and maintain the life quality of patient. In contrast, late-stage CKD often results in a diminished quality of life and prognosis, and end-stage CKD is linked with increased morbidity, mortality, and healthcare costs. Comprehensive care for CKD patients necessitates a multidisciplinary approach to mitigate disease progression, prevent complications, and if necessary, help patients make timely preparation for kidney replacement therapy (KRT) that align with their preferences.

Although the eGFR-based CKD staging is routinely used for making diagnosis and prognosis decisions, it has been shown to be inadequate due to the significant heterogeneity in patients of the same stages and the variability in their disease progression^3–5^. For example, some patients with early-stage CKD never progress while others may experience rapid disease progression. The clinical strategies for these patients should differ to avoid under- or over-treatment despite they are categorized to the same stage. For the following reasons, it is crucial to develop an accurate prediction model for the disease progression in CKD patients. Firstly, early identification of high-risk CKD can enable both patients and healthcare providers to actively manage CKD and its associated risk factors^6–8^, such as diabetes or hypertension. High-risk patients can promptly receive the latest albeit more expensive treatments available to reduce CKD progression. Moreover, this group may also benefit from earlier nephrology consultations and education regarding KRT options. Secondly, the identification and management of high-risk early-stage CKD is particularly important for racial and ethnic minority groups as they face a higher risk of CKD progression and related complications^9,10^. Thirdly, a prediction model that gauges the risk of CKD progression can also benefit patients at a low risk as well. When elderly individuals are diagnosed with CKD but are deemed to have a low progression risk, some are more likely to succumb to age-related conditions or other co-morbidities before the CKD advances^11^. In such scenarios, physicians may be reluctant to screen for CKD or even inform patients to avoid unnecessary worry or overaggressive treatments. Identifying these patients can lead to improved health outcomes and a better quality of life, as they can avoid the financial strain and potential side effects of unnecessary medications, as well as the drawbacks of aggressive blood pressure and glycemic control^12–15^.

To address these challenges, we have developed a novel data processing pipeline and an effective artificial intelligence (AI) model to predict CKD progression from early stages to late stages. Specifically, we predict whether a patient will progress from CKD stages II/III to stages IV/V within a defined timeframe based on longitudinal electronic health records (EHRs). A data assembly and processing pipeline has been developed to convert patient EHRs into well-structured time series data. This data encompasses a range of clinical variables, including demographics, vital information, lab tests, and health-related behaviors. Building upon the time series data, we have constructed recurrent neural network (RNN) models with long short-term memory (LSTM) units^16,17^ to predict CKD progression. Additionally, through feature selection analysis, we have also identified the clinical variables that exhibit the highest predictive capability concerning CKD progression.

Most existing methods for predicting CKD progression have primarily concentrated on predicting the advancement towards end-stage kidney disease (ESKD) or kidney failure as indicated by the necessity for KRT like dialysis or kidney transplantation^5,18–26^. Static Cox proportional hazards models have been used to predict CKD progression from stages III/IV/V to kidney failure^5,18^. These models used either four or eight demographic and lab-test variables collected at a single time point to make predictions, ignoring temporal information embedded in longitudinal EHRs. As an enhancement, a dynamic Cox proportional hazards models was subsequently introduced. This model treated certain variables as time-dependent covariates by utilizing their values from all previous clinical visits^19^. Another approach employed Cox regression with feature selection through the least absolute shrinkage and selection operator (LASSO) technique. It was applied to predict kidney failure based on cross-sectional patient data^20^. Furthermore, an additional Cox model was employed to identify the association between systolic blood pressure and a composite outcome of CKD progression, which encompassed a ≥ 50% decrease in eGFR from the baseline and ESKD^21^. Temporal abstraction techniques have been utilized to extract features from EHR time series for predicting progression to ESKD^23^. In addition to disease progression, predictive modeling has targeted certain CKD prognosis markers, including patient mortality and kidney transplant failure^27–29^. Moreover, multiple conventional machine learning methods have been used to predict the onset of KRT^24^.

Compared with existing predictive approaches for CKD progression, our proposed method takes a step forward in several aspects. Firstly, our prediction target—the transition from stages II/III to stages IV/V—remains a relatively underexplored area. Most previous investigations have centered around predicting kidney failure or ESKD. An accurate prediction of CKD progression from an early stage to a late stage holds the potential for early risk detection and facilitates timely medical interventions to potentially halt disease advancement during its initial phases. Given the shortage of nephrologists and the emergence of costly new medications to prevent CKD progression, knowing which patients require early nephrology referrals and more intensive treatment plans can reduce healthcare costs and unnecessary medication burdens. Secondly, in contrast to the prevalent utilization of cross-sectional observations in existing methods, we leverage longitudinal data for predicting CKD progression. Considering the chronic nature of CKD, longitudinal EHRs over time are expected to provide a more comprehensive depiction of the entire path of disease history, thus yielding richer information for prognostication. To effectively utilize longitudinal EHRs, we have built a novel data processing pipeline to process patient EHRs and transform them into well-structured time series for subsequent modeling analysis. The pipeline integrates various variables of demographics, lab tests, vital information, and health behaviors, imputes their values as necessary, and converts them into feature vectors at sequential time points. Thirdly, to the best of our knowledge, our study represents the pioneering use of LSTM RNN for predicting CKD progression. Previous related works have predominantly relied on Cox proportional hazards models and conventional machine learning techniques. The LSTM RNN model can capture temporal relationships embedded within complex data structures and excels in forecasting future events based on time course data^16,17,30–33^. Our analysis results demonstrate that it outperforms existing methods, indicating the advantage of using longitudinal EHRs and LSTM RNN for predicting CKD progression. Lastly, but not the least, our study shows that the proposed RNN model achieves both high and stable accuracy in predicting CKD progression using the time-course measurements of a single clinical variable, eGFR. While previous studies employed multiple clinical variables for such predictions^5,18–20,23,26^, the simplicity of our approach, demanding information of only one clinical variable, makes its practical applications feasible and straightforward.

## Methods

### Retrieval, assembly, and processing of EHRs

This study is approved by the institutional review board (IRB) of the University of Chicago under IRB number 19-1151. All methods in this study have been performed in accordance with the relevant guidelines and regulations. Informed consent is waived by the IRB of the University of Chicago because of the retrospective nature of the study with minimal risk to participants. Longitudinal patient EHRs from 01/01/2006 to 07/01/2019 are retrieved from the University of Chicago Medical Center. We include all patients who have experienced at least one stable stage II/III period, defined as having two eGFR values $\ge 30$ and $\le 89$ that are at least 90 days apart and that any eGFR value between them must also fall within this range. A total of 82,667 patients meets the criterion. For each patient, the extracted EHRs include four types of clinical variables including demographics, vital information, lab tests, and health behaviors. We choose clinical variables that are likely to be available and can potentially be linked to CKD progression. Table 1 lists all the variables in the retrieved data. These variables are associated with major CKD risk factors and co-morbidities that may convey additional risks. Transformations have been performed for some variables, which are detailed in Sect. 1 of the Supplementary Information. Predictive models are constructed using two sets of variables: all variables (see Table 1) and essential variables (indicated by italic font in Table 1). All demographics and vital information variables are included in the essential set. Eight lab test variables with the highest frequency are also included in the essential set. In the case of health behavior variables, only the primary indicators of drug, alcohol, and smoking usage are part of the essential set, whereas variables specifying the types of drugs and smoking are excluded. These essential variables are mainly associated with CKD risk factors.

**Table 1:** EHR variables collected for CKD progression prediction.

Variable category	Variable name
Demographics	*Sex, race*
Vital information	*Systolic and diastolic blood pressures, body mass index, oxygen saturation, height, weight*
Lab test	*Albumin, blood urea nitrogen, calcium, creatinine*, ferritin, HbgA1C, *Hgb*, cholesterol, LDL, HDL, parathyroid hormone, *phosphorous*, saturation, triglycerides, uric acid, urine creatinine, urine micro albumin, urine protein, urine RBC count, vitamin D25, *bicarbs CO2, eGFR*
Health behavior	*Illegal drug usage status*, indicators of injection, IV, pill, and marijuana usages *Alcohol usage status* *Smoking status*, indicators of cigarette, cigar, and tobacco usages

The italic variables form the essential set.

In our study, all patients were at stages II/III at the beginning of their longitudinal EHRs. Some patients later progressed to stage IV/V and are considered cases. In contrast, the controls are those who never progressed to late stages by the end of the study time (07/01/2019). To qualify as a case, a patient must have experienced at least one stable stage IV/V period, as indicated by having two eGFR values ≤ 29, separated by at least 90 days, and with any eGFR value in between also being ≤ 29. For case patients, the start time of the first stable stage IV/V period is taken as the transition point to the late stage, denoted as ${T}_{{\text{tra}}}$. Case patients are also required to have a minimum of four eGFR values, with the first two values not belonging to a stable stage IV/V period. Control patients, on the other hand, do not have any stable stage IV/V period and are also required to have at least four eGFR values. Based on these selection criteria, 1,968 case patients and 70,877 control patients have been identified.

**Figure 1:**
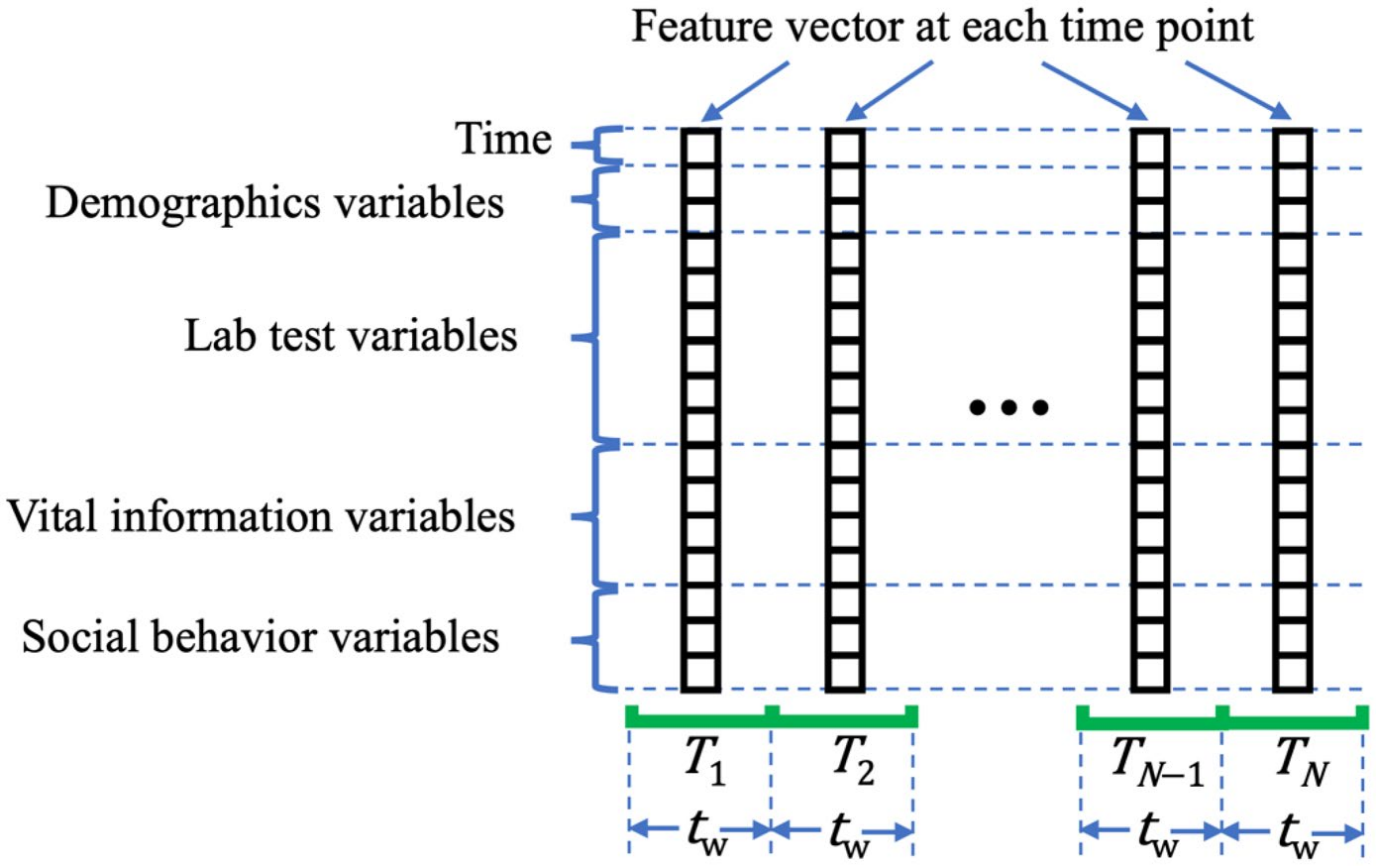
Illustration of assembling a time series of feature vectors based on EHRs.

The longitudinal EHRs of each patient are transformed into time series, structured as feature vectors at sequential time points. Figure 1 provides an illustration of a patient’s time series. The time points are denoted as ${T}_{1}, \dots , {T}_{N}$, each representing a time interval of ${t}_{{\text{w}}}$ days (e.g., ${t}_{{\text{w}}}=7$). ${T}_{n}$ is the midpoint of the *n*th time interval, where $n\in \left\{1,\dots ,N\right\}$. At each time point, a feature vector is constructed, comprising the values of all variables. ${T}_{n}$ is also included in the vector as an additional feature. If multiple readings of a lab test or vital information variable are recorded within a time interval, their average is used to create the feature vector. For health behavior variables, the latest reading in a time interval is used in the feature vector. Please refer to Sect. 2 of the Supplementary Information for more details on constructing time series and imputing missing values.

**Figure 2:**
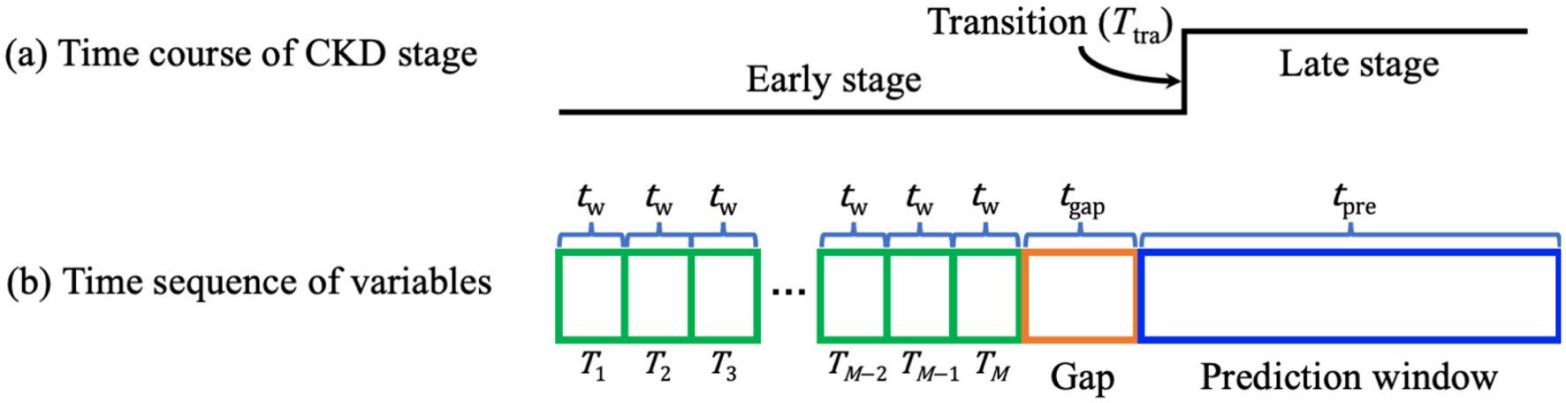
Illustration of time series data of a case patient.

In our analysis, we focus on the prediction of CKD progression from early to late stages within a specific time period denoted as ${t}_{{\text{pre}}}$ (e.g., 90 or 365 days). For a case patient, only feature vectors representing patient information before the time ${T}_{{\text{tra}}}-{t}_{{\text{gap}}}$ are included in the analysis, where ${t}_{{\text{gap}}}$ is the length of a gap period (e.g., 7 days). See Fig. 2 for an illustration of ${t}_{{\text{gap}}}$, which is inserted between the prediction window (indicated by the blue block in Fig. 2b) and the time points used for predictive modeling (indicated by green blocks in Fig. 2b). The gap period is introduced to prevent potential cases where CKD progression is determined based on lab results measured at the last time point (*T*_*M*_ in Fig. 2b). In addition, we require that valid case patients must have the transition point falling within the prediction window and have at least five time points in their time series. If a patient’s record contains more than 100 time points, only the latest 100 time points are used for analysis. For control patient time series, we remove the latest feature vectors spanning a period of ${t}_{{\text{pre}}}$ to ensure that the remaining feature vectors correspond to a disease process that will not progress during ${t}_{{\text{pre}}}$. To create a dataset for predictive analysis, we match each case patient with four control patients based on patient race, sex, age, and time series length whenever sufficient qualified control patients are available. Please refer to Sect. 3 in the Supplementary Information for detailed information about matching case and control patients. Supplementary Table 1 summarizes the matched data generated using all variables, with ${t}_{w}=7$ days, ${t}_{{\text{gap}}}=7$ days, and ${t}_{{\text{pre}}}=365$ days. Supplementary Fig. 1a shows the distribution of the time series length of case patients, with a peak at 100 due to the truncation of long patient time series to 100 time points. The control patient sequence length follows the same distribution (not shown) because each case patient is matched with four control patients of the same sequence length. Supplementary Fig. 1b shows the distribution of the time duration between the transition point and the last time point of feature vectors used in the analysis for case patients. The distribution shows a peak at the lower end but also has a long thick tail extending beyond 100 days.

Stage III CKD can be further divided into two sub-stages: stage IIIa (45 ≤ eGFR ≤ 59) and stage IIIb (30 ≤ eGFR ≤ 44). A more challenging task is predicting the progression of patients from Stage II or Stage IIIa to Stages IV and V. This prediction would enable the earlier detection of high-risk patients before they reach stage IIIb. To apply our modeling approach to this task, we have generated data while excluding patient information in stage IIIb. For more details about the data generation process, please refer to Sect. 4 of the Supplementary Information.

As a result of the data preprocessing, we generate multiple datasets that include matched case and control patients. These datasets may or may not include patient stage IIIb information and may contain all variables or only essential variables. We also create datasets with multiple prediction window sizes. All these datasets are generated using ${t}_{w}={t}_{{\text{gap}}}=7$ days. Supplementary Table 2 provides information on the number of case and control patients in the matched data generated under different settings. It is worth noting that excluding stage IIIb data significantly reduces the number of valid case and control patients. To prepare the data for predictive modeling, we assign a binary classification label to each patient. Patients whose CKD condition progresses to stages IV/V within the prediction window (i.e., case patients) receive a label of 1, while patients whose CKD condition does not progress to stages IV/V within the prediction window (i.e., control patients) receive a label of 0.

### Construction and evaluation of LSTM RNNs and baseline prediction models

**Figure 3:**
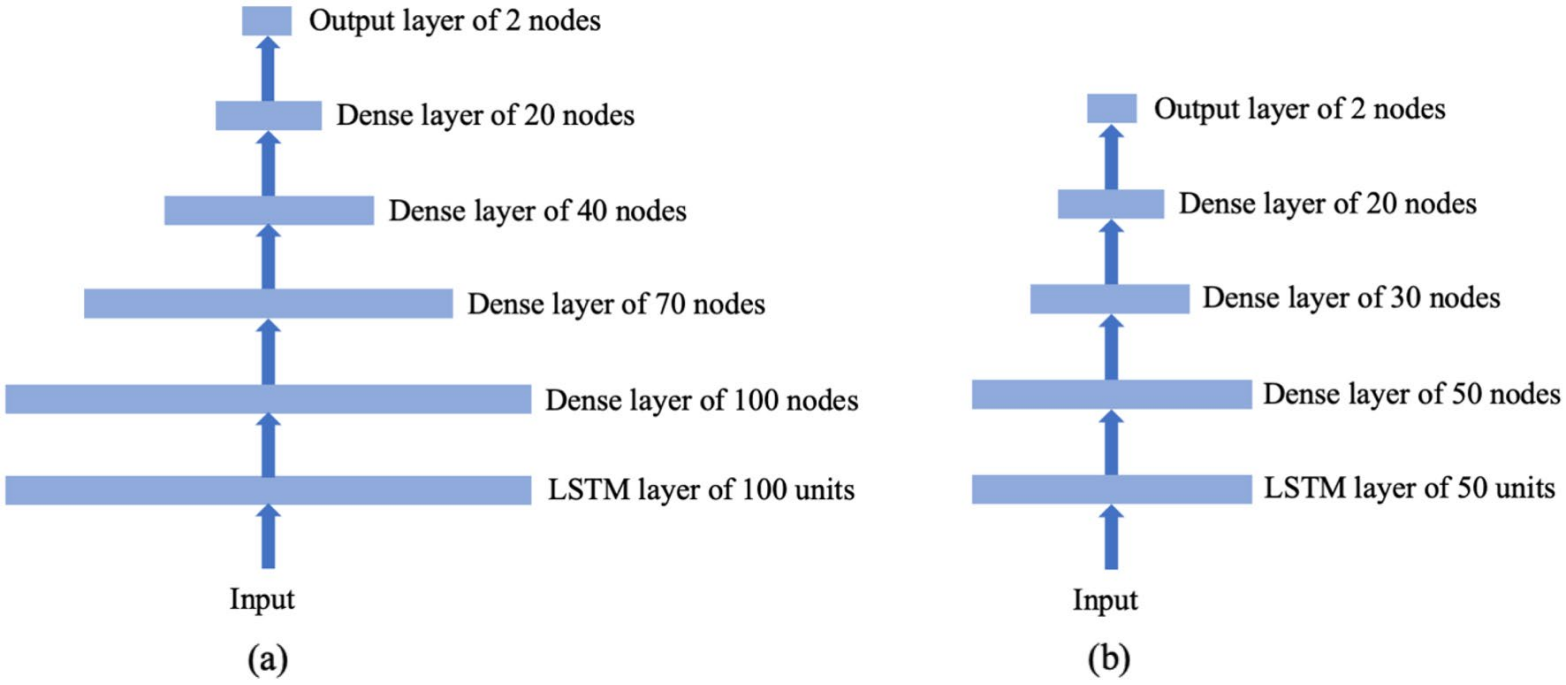
Architectures of LSTM RNN models used in the analysis. (**a**) Model architecture used for analysis based on all variables. (**b**) Model architecture used for analysis based on essential variables.

Figure 3 shows the architecture of two LSTM RNNs used for modeling using either all variables or essential variables only. Both models consist of a single LSTM layer and multiple dense layers. The model that utilizes only essential variables has fewer dense layers and nodes compared with the model using all variables, due to the reduced number of input features for essential variables. All dense layers in both models use ReLU activation functions and dropout mechanisms. The dropout rate remains the same across all dense layers within a model, which is the only hyper-parameter subject to tuning during model training. The dropout rate is selected from [0, 0.1, 0.2, 0.3, 0.4, 0.5, 0.7]. The output layer comprises two nodes corresponding to the two classes in the data, i.e., case and control patients. It employs the softmax activation function, ensuring that the output values of the two nodes sum to one and form probabilistic prediction outcomes. The weighted cross-entropy loss function is used for model training. In this context, the weight for control patients is set to 1, while the weight for case patients is determined by the sample size ratio between control patients and case patients in the training set. We conduct tenfold cross-validation for training and evaluating the prediction models. Eight data folds are used for training the prediction model, one for validation to select the dropout rate and implement early stopping to avoid overfitting, and one for testing. The testing data fold is used to assess prediction performance using the area under the receiver operating characteristic curve (AUROC), the area under the precision-recall curve (AUPRC), and the Matthew’s correlation coefficient (MCC) as performance metrics. AUROC evaluates the accuracy of ranking case patients above control patients based on prediction results. AUPRC has been demonstrated to be more informative when evaluating prediction performance on imbalanced data^34^. MCC provides a balanced assessment of type I and type II errors following classification through thresholding the probabilistic prediction outcomes using a cutoff of 0.5. A total of 100 random cross-validation trials are performed. See Sect. 5 in the Supplementary Information for a detailed explanation of LSTM RNN model implementation and training.

For comparison, we also implement the static and dynamic Cox proportional hazards models, which are widely used as a standard in practice^5,18,19^. In both models, the time-to-CKD progression is used as the response variable, and a Cox model is applied to regress the time-to-CKD progression on a set of covariates. Regression coefficients are estimated via the maximum likelihood estimation. These coefficients are used to compute the estimated hazard function for each patient by plugging in the patient’s covariate values. The estimated hazard function is then used to compute the survival function at any future time $T$ for the patient, which is the probability of CKD progression at time $T$. By applying a probability threshold, we can predict the binary status of CKD progression at time $T$ for the patient. In the static Cox model, the covariate values at the first time point are used to fit the model, following the approach used in the original publication^5^. Conversely, the dynamic Cox model incorporates both time-dependent and time-independent covariates^19^. Demographics variables are taken as time-independent covariates, while all other variables are taken as time-dependent covariates. For time-dependent covariates, their longitudinal time-course measurements are used to fit the dynamic Cox model, a crucial distinction from the static Cox model. Estimation of the dynamic model is conducted through maximum likelihood, and the input data must be organized in a counting-process style^35^. For predicting CKD progression under the dynamic model, the process remains similar to the static Cox model, except that the dynamic model incorporates values of time-dependent covariates at sequential time points. Both Cox models are trained and evaluated using the same cross-validation data partitions as those used for the analysis of LSTM RNNs. However, the training and validation sets are combined for fitting the Cox models since there is no requirement for hyperparameter tuning or early stopping of model training. The prediction performance of Cox models is evaluated on the testing set using the C-statistic (equivalent to AUROC), AUPRC, and MCC. A 0.5 cutoff is applied on the predicted probability of CKD progression to calculate the MCC. Our implementation of the Cox proportional hazards models follows the methodologies detailed in the original publications^5,18,19^, which provide more information about the methodology.

We also include two conventional machine learning methods, namely random forest^36^ and LightGBM^37^, as baseline models for comparison. Random forest constructs multiple decision trees on random subsets of data and employs the average of their predictions as the final outcome^36^. LightGBM is a fast gradient boosting decision tree algorithm that uses techniques of gradient-based one-side sampling and exclusive feature bundling to expedite model training^37^. Both of these models are trained using patient data at the latest time point to predict the progression risk. We use the same data partitions of training, validation, and testing sets as those used in the analysis of LSTM RNNs to perform cross-validation evaluations for random forest and LightGBM. For random forest, the training set and the validation set are combined for model training, as no hyperparameter tuning or early stopping of model training is necessary. AUROC, AUPRC, and MCC measurements are calculated to evaluate the prediction performance. A cutoff of 0.5 is applied on the probabilistic prediction outcome to compute MCC. See Sect. 5 in the Supplementary Information for more details of implementing and training random forest and LightGBM models.

## Results

### Evaluation and comparison of prediction performance

We conduct an evaluation and comparison of prediction performance between LSTM RNN and baseline methods across two distinct prediction windows: 365 days and 90 days. Table 2 shows the mean and standard deviation of prediction performance derived from cross-validation trials. In all different settings of prediction window and variable usage, LSTM RNNs consistently achieve an average AUROC exceeding 0.95, accompanied by a standard deviation of less than 0.01, which indicates a high and robust prediction performance. Moreover, the obtained average AUPRC always exceeds 0.83, while the average MCC always exceeds 0.72, further affirming the accuracy of classification outcomes. Importantly, in all analysis schemes, our proposed LSTM RNN models consistently outperform all baseline methods evaluated using all three performance metrics. The only exception is that the dynamic Cox proportional hazards model achieves the same average prediction performance as the LSTM RNN model does for predicting disease progression within 90 days when evaluated using AUPRC. Comparing the use of all variables against only essential variables, employing all variables yields a better prediction performance for LSTM RNN and the two Cox proportional hazards models, but not for random forest and LightGBM. Interestingly, the performance enhancement achieved by LSTM RNNs over the Cox models is more pronounced in the context of the 365-day prediction window compared to the 90-day prediction window. Between the two Cox proportional hazards models, the dynamic model exhibits superior prediction performance relative to the static model. This observation aligns with prior findings suggesting that longitudinal EHRs furnish more predictive information than cross-sectional data for CKD progression prediction^19^. Furthermore, the prediction performances of random forest and LightGBM surpass those of both static and dynamic Cox proportional hazards models when evaluated using AUROC and MCC.

**Table 2:** Comparison on prediction performance of LSTM RNN and baseline methods.

Prediction window	Model	All variables			Essential variables		
		AUROC	MCC	AUPRC	AUROC	MCC	AUPRC
365 days	LSTM RNN	0.967 (0.006)	0.766 (0.024)	0.878 (0.023)	0.964 (0.008)	0.756 (0.029)	0.861 (0.029)
	Random forest	0.940 (0.008)	0.659 (0.034)	0.802 (0.029)	0.953 (0.009)	0.713 (0.031)	0.835 (0.026)
	LightGBM	0.941 (0.008)	0.677 (0.030)	0.813 (0.026)	0.953 (0.008)	0.720 (0.027)	0.840 (0.026)
	Dynamic Cox proportional hazards model	0.837 (0.043)	0.118 (0.018)	0.691 (0.009)	0.836 (0.042)	0.111 (0.020)	0.682 (0.010)
	Static Cox proportional hazards model	0.732 (0.044)	0.062 (0.017)	0.687 (0.010)	0.704 (0.051)	0.045 (0.015)	0.678 (0.011)
90 days	LSTM RNN	0.961 (0.009)	0.744 (0.032)	0.860 (0.033)	0.957 (0.008)	0.723 (0.034)	0.838 (0.035)
	Random forest	0.937 (0.012)	0.644 (0.040)	0.801 (0.033)	0.946 (0.011)	0.678 (0.045)	0.816 (0.040)
	LightGBM	0.936 (0.012)	0.673 (0.041)	0.806 (0.031)	0.944 (0.011)	0.697 (0.040)	0.814 (0.041)
	Dynamic Cox proportional hazards model	0.904 (0.033)	0.146 (0.030)	0.858 (0.011)	0.900 (0.043)	0.142 (0.023)	0.838 (0.012)
	Static Cox proportional hazards model	0.782 (0.059)	0.089 (0.020)	0.855 (0.013)	0.716 (0.061)	0.077 (0.020)	0.836 (0.013)

Numbers before and in the parentheses are mean and standard deviation, respectively.

For the more challenging task of predicting CKD progression with the exclusion of stage IIIb data, we explore prediction windows spanning 365 days (1 year), 1095 days (3 years), and 1825 days (5 years). Table 3 shows the mean and standard deviation of prediction performance obtained through cross-validation. Our proposed LSTM RNN consistently achieves an average AUROC of around 0.9, an average MCC surpassing 0.53, and an average AUPRC over 0.68, across different prediction window lengths and variable utilization scenarios. Its performance remains comparable whether employing all variables or essential ones exclusively. Importantly, LSTM RNNs consistently outperform all baseline methods across different configurations of prediction windows and variable usage, as evidenced by its higher average AUROC, MCC, and AUPRC measurements. Compared with the previous prediction task that incorporates stage IIIb data, the performance of all models drops, likely due to the more difficult prediction task and the constraint posed by the smaller dataset available for modeling analysis (Supplementary Table 2). Between the two Cox models, the dynamic model once again generally outperforms the static model. Comparison between random forest and LightGBM reveals that random forest achieves higher average AUROC and AUPRC, while LightGBM achieves a better average MCC. This discrepancy potentially indicates their different capabilities in rank-ordering case and control patients, as well as in making classification predictions.

**Table 3:** Comparison on prediction performance of LSTM RNN and baseline methods when stage IIIb data are excluded.

Prediction window	Model	All variables			Essential variables		
		AUROC	MCC	AUPRC	AUROC	MCC	AUPRC
365 days	LSTM RNN	0.887 (0.041)	0.531 (0.103)	0.701 (0.094)	0.889 (0.038)	0.536 (0.100)	0.688 (0.094)
	Random forest	0.865 (0.041)	0.419 (0.119)	0.650 (0.096)	0.883 (0.038)	0.424 (0.101)	0.676 (0.083)
	LightGBM	0.848 (0.044)	0.475 (0.104)	0.644 (0.092)	0.865 (0.044)	0.488 (0.113)	0.653 (0.091)
	Dynamic Cox proportional hazards model	0.557 (0.165)	0.049 (0.077)	0.371 (0.075)	0.486 (0.081)	− 0.027 (0.013)	0.075 (0.034)
	Static Cox proportional hazards model	0.517 (0.127)	0.020 (0.065)	0.367 (0.139)	0.482 (0.082)	− 0.017 (0.060)	0.072 (0.035)
1095 days	LSTM RNN	0.912 (0.022)	0.598 (0.059)	0.730 (0.054)	0.897 (0.025)	0.562 (0.056)	0.703 (0.057)
	Random forest	0.886 (0.023)	0.448 (0.068)	0.671 (0.062)	0.887 (0.024)	0.459 (0.077)	0.679 (0.066)
	LightGBM	0.878 (0.023)	0.531 (0.059)	0.670 (0.055)	0.875 (0.027)	0.515 (0.063)	0.663 (0.066)
	Dynamic Cox proportional hazards model	0.600 (0.095)	0.065 (0.067)	0.459 (0.038)	0.574 (0.114)	0.018 (0.078)	0.453 (0.034)
	Static Cox proportional hazards model	0.568 (0.091)	0.044 (0.071)	0.458 (0.037)	0.503 (0.097)	0.017 (0.079)	0.447 (0.033)
1825 days	LSTM RNN	0.913 (0.018)	0.609 (0.049)	0.745 (0.051)	0.904 (0.020)	0.594 (0.056)	0.719 (0.052)
	Random forest	0.881 (0.024)	0.425 (0.075)	0.654 (0.063)	0.887 (0.022)	0.478 (0.061)	0.691 (0.046)
	LightGBM	0.872 (0.027)	0.506 (0.069)	0.653 (0.064)	0.880 (0.021)	0.529 (0.047)	0.680 (0.048)
	Dynamic Cox proportional hazards model	0.633 (0.095)	0.071 (0.099)	0.571 (0.020)	0.577 (0.102)	0.040 (0.080)	0.567 (0.021)
	Static Cox proportional hazards model	0.610 (0.095)	0.051 (0.073)	0.569 (0.022)	0.575 (0.103)	0.028 (0.077)	0.559 (0.021)

Numbers before and in the parentheses are mean and standard deviation, respectively.

The results presented in Tables 2 and 3 show that our LSTM RNN model performs better when the prediction window becomes larger. One possible explanation for this trend is that with a larger prediction window, a greater number of valid case patients can be identified and included in the analysis. Consequently, more control patients can be matched with the case patients and included in the analysis. Supplementary Table 2 illustrates that an increased amount of data is available for predictive modeling as the prediction window size increases. As a result, the predictive performance of LSTM RNNs improves.

### Variable selection and importance evaluation

The results in Tables 2 and 3 show that the prediction performance of LSTM RNN remains similar or slightly improved when using all variables compared to using only the essential variables. This leads to two subsequent questions for further investigation. (1) What prediction performance can be achieved using even fewer variables? (2) What are the most important features? To answer these questions, we focus on the prediction task with a 365-day prediction window and stage IIIb data included. We apply a sequential forward variable selection procedure to identify the most predictive variables. This procedure begins by identifying the most predictive single variable, followed by the incremental addition of one variable at a time to the model. At each step, an exhaustive search is applied to identify the variable that, when added, yields the highest prediction performance. Time is always included as an input feature since our prediction is based on time series data. For a detailed description of the variable selection procedure, please refer to Sect. 6 of the Supplementary Information.

**Figure 4:**
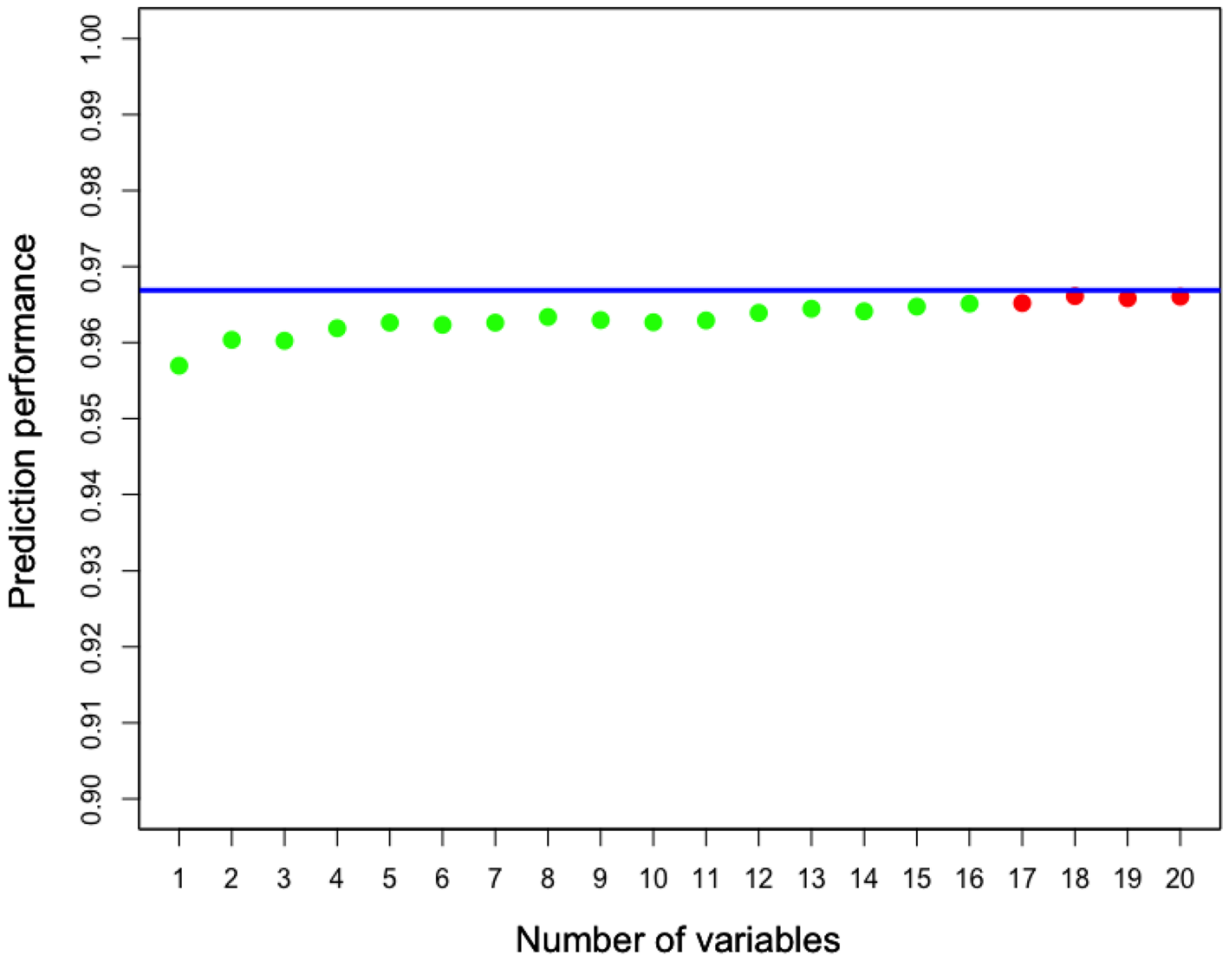
Prediction performance of using different numbers of variables identified in the sequential forward search. Dots indicate the average AUROC obtained using selected variables across cross-validation trials. The blue line indicates the average AUROC obtained using all variables. Green indicates that the performance difference between selected variables and all variables is statistically significant (adjusted p-value $\le$ 0.05), while red indicates the performance difference is not statistically significant (adjusted p-value > 0.05).

Figure 4 shows the average AUROC of models built using different numbers of variables and evaluated via cross-validation. Notably, eGFR is consistently identified as the most predictive single variable in 100% of the cross-validation trials (see Supplementary Table 3). It achieves an average AUROC of 0.957 with a standard deviation of 0.009 in cross-validation, indicating a high and stable accuracy when using this single variable. The prediction performance increases slightly as additional variables are added, eventually reaching a level of accuracy comparable to that achieved by the model employing all variables (as indicated by blue line in Fig. 4). To assess the difference in prediction performance between selected variables and all variables, we employ a two-sided pairwise t-test. The obtained p-value is adjusted for multiple tests using the Benjamini–Hochberg method^38^. Our analysis reveals that the performance difference is statistically significant (adjusted p-value $\le$ 0.05) when utilizing no more than 16 variables (as illustrated in Fig. 4). Supplementary Table 3 shows the frequencies of variable selection across cross-validation trials during the sequential forward search process. When selecting two variables for prediction, smoking status emerges as the second most frequently selected variable, with a frequency of 26.67%, which is significantly lower than the 100% selection frequency of eGFR. In summary, longitudinal eGFR is the best predictor enabling our models to achieve a high level of accuracy. The inclusion of additional variables yields only a marginal improvement in predictive accuracy.

### Race-specific prediction performance

We divide the patient cohort into three racial groups, including African American, white, and others, to investigate the variation in prediction performance among these racial groups. We calculate the prediction performance (AUROC) for each racial group in all analysis schemes. Table 4 presents the mean and standard deviation of AUROC values across cross-validation trials for each racial group. The maximum performance difference between racial groups is also calculated and shown in Table 4. In the analyses that include stage IIIb data, the difference in average AUROC between racial groups is $\le$ 0.008, indicating only small variations among races. In the analyses without stage IIIb data, the difference in average AUROC between racial groups is $\le$ 0.064. The bigger difference in prediction performance is probably due to the smaller sample size when stage IIIb data are excluded, which may lead to increased variation between racial groups due to random effects in small sample size cases.

**Table 4:** Race-specific prediction performance and its difference among racial groups.

Variable	Stage IIIb data excluded	Prediction window (in days)	Prediction performance (AUROC)			Maximum performance difference between groups
			African American	White	Others	
All	No	90	0.962 (0.010)	0.959 (0.019)	0.965 (0.036)	0.006
		365	0.966 (0.006)	0.972 (0.013)	0.972 (0.031)	0.006
	Yes	365	0.887 (0.050)	0.882 (0.083)	0.938 (0.079)	0.056
		1095	0.914 (0.025)	0.898 (0.044)	0.958 (0.062)	0.060
		1825	0.911 (0.023)	0.911 (0.038)	0.965 (0.047)	0.054
Essential	No	90	0.957 (0.010)	0.956 (0.023)	0.962 (0.049)	0.005
		365	0.962 (0.009)	0.970 (0.012)	0.967 (0.033)	0.008
	Yes	365	0.891 (0.047)	0.866 (0.095)	0.920 (0.081)	0.054
		1095	0.893 (0.030)	0.899 (0.048)	0.956 (0.074)	0.064
		1825	0.901 (0.024)	0.909 (0.039)	0.939 (0.057)	0.038

For prediction performance, numbers before parentheses are average AUROC and numbers in parentheses are standard deviations. The maximum prediction performance difference is the largest difference in average AUROC among three pairwise comparisons, i.e., African American vs. white, African American vs. others, and white vs. others.

## Conclusion and discussion

We have introduced a novel approach for predicting the progression of CKD from stages II/III to stages IV/V, based on longitudinal patient EHRs. Our model can identify high-risk patients using commonly available clinical variables. This method combines an EHR preprocessing pipeline with an AI predictive model, collectively yielding a commendable accuracy in forecasting CKD progression. The EHR preprocessing steps integrate various clinical variables and convert them into time series data suitable for modeling using RNNs. Our analysis reveals that using the time series of a single variable, eGFR, the RNN model achieves an average AUROC of 0.957 for predicting disease progression within a year. With the inclusion of additional clinical variables for prediction, the average AUROC increases to 0.967. In both scenarios, the standard deviation of AUROC across cross-validation trials remains below 0.01, indicating a consistently high prediction performance. Our models outperform existing methods, including static and dynamic Cox proportional hazards models, random forest, and lightGBM, assessed by the AUROC, AUPRC, and MCC performance metrics. When stage IIIb data are excluded to simulate the challenge of early detection of high-risk patients, our proposed models maintain an average AUROC hovering around 0.9.

Compared with existing methods for predicting CKD progression, our approach offers advantages in three aspects. Firstly, we utilize longitudinal patient EHRs to predict CKD progression, in contrast to the prevalent use of cross-sectional observations in most previous studies. Longitudinal EHRs provide more abundant information regarding disease progression than snapshot data. To support effective disease modeling based on longitudinal EHRs, we have developed a novel data processing pipeline to process and integrate various EHR variables, converting them into well-structured time series data suitable for subsequent modeling analysis. It is worth noting that this EHR processing pipeline is not specific to the task of CKD progression prediction. It is a general framework applicable to processing any longitudinal EHR data of demographics, vital information, lab tests, and health behaviors, for various analysis purposes. Secondly, to the best of our knowledge, our work is the first attempt of using LSTM RNNs for predicting CKD progression. Existing related works usually use Cox proportional hazards models and conventional machine learning methods for predictions. LSTM RNNs are powerful tools for detecting temporal relationships and making predictions about future events. Our analysis results, as presented in Tables 2 and 3, demonstrate the superior performance of LSTM RNNs over other baseline methods across various CKD progression prediction scenarios. Thirdly, our variable selection analysis reveals that LSTM RNNs constructed on the longitudinal records of a single clinical variable, eGFR, can achieve an average AUROC of 0.957. In contrast to previous studies that rely on multiple clinical variables for predicting CKD progression^5,18–20,23,26^, the simplicity of our approach, demanding information of only one clinical variable, renders its adoption for practical purposes both achievable and straightforward.

Our work achieves a high accuracy for predicting CKD progression from stages II/III to stages IV/V, which is of paramount importance to patient care. Our prediction target is CKD progression from early stages to late stages, which is underexplored in previous works. The majority of existing works within this domain have primarily concentrated on predicting progression to end-stage kidney disease or kidney failure^5,18–25^. Our study holds the potential to significantly enhance early detection of high-risk patients, facilitating timely medical interventions to potentially stop disease advancement in its initial phases. All patients with CKD should receive guideline recommended care such as urine testing for proteinuria, blood pressure control, angiotensin blockade, and testing for glycemic control. Patients with high risk of progression to late stages can be started earlier on newer medications proven to reduce CKD progression, like SGLT-2 inhibitors, finerenone, and GLP-1 agonists. Despite their proven efficacy, these medications are often underutilized due to elevated costs or clinical inertia^39–41^. An accurate prediction of CKD progression to late stages not only refines risk stratification, but also provides evidences for clinicians to initiate high-risk patients on these medications earlier, given the anticipated greater benefit^42^. Such predictions may motivate patients at a high risk of CKD progression to persist with these new medications, despite potential regimen complexities, side effects, and higher co-pays^43^. This can also encourage patients to make lifestyle changes essential for reducing the risk of CKD progression^44^. Lastly, given a shortage of nephrology appointments, primary care clinicians can leverage the predicted risk to decide whether to refer patients to nephrology early or delay referral^45,46^. With an accurate prediction of CKD progression, physicians can avoid making ad hoc clinical decisions that can result in either insufficient care for those who will ultimately progress to more advanced CKD or unnecessary treatments and costs for patients who will not progress. Accurate prediction of disease progression risk could significantly facilitate individualized decision making, enabling early and appropriate patient care, and substantially reduce healthcare costs.

A major advantage of the EHR dataset used in our analysis is its enriched population of minority patients, especially African Americans. Some previous prediction analyses had a low representation of African Americans despite their higher risk of CKD progression^5,18,20^. This distinctive characteristic introduces a novel perspective on the potential application of the proposed model within these specific patient populations. However, the limitation inherent to utilizing only one dataset as an illustration lies in the absence of validation across additional patient cohorts. To address this limitation, our future endeavors will involve collecting more EHR data of CKD patients, including data of other racial groups, to make our models more representative and evaluate the generalizability of our prediction models to other patient cohorts.

Additional future work has been considered and planned to follow the current study. Firstly, other types of EHRs can be added for predictive modeling, such as patient diagnostic ICD (International Classification of Diseases) codes, procedures, medications, and clinical notes, which may provide additional power for modeling disease progression. The utilization of embeddings for ICD codes, procedure codes, medications, and clinical notes, as explored by prior research^30,32,33,47–51^, can be adapted to enhance our neural network models. Secondly, the proposed EHR processing pipeline and prediction model need to be integrated into the EHR system at medical centers for an ideal application environment. This integration would facilitate real-time predictions, thereby providing valuable decision support for patient care. Thirdly, to validate the clinical efficacy and prediction capabilities of our model, a prospective study, such as a clinical trial, is imperative. Such an endeavor would substantiate the practical utility of our model and its potential benefits for patient care. Fourthly, the proposed data processing and modeling approach can also be extended to other prediction targets, such as kidney failure and patient mortality. Relevant data need to be collected, and the data processing pipeline and prediction models need to be implemented for predicting these prognosis and progression markers. Lastly, leveraging the predicted CKD progression risks and patient medical records, we can develop methods to identify patients who have received insufficient care or unnecessary treatments. After identifying these patients, the influence of risk prediction performance on their population size can also be investigated.

### Supplementary Information

Supplementary Information.

## Data Availability

The dataset used in this study is not publicly available due to the proprietary nature of the data and patient privacy concerns. Interested researchers should contact the corresponding authors to inquire about the access. A data use agreement and institutional review board approval will be required as appropriate.
