# Supplementary material for: Prediction of chronic kidney disease progression using recurrent neural network and electronic health records
Source: Sci Rep. 2023 Dec 13;13:22091. doi: 10.1038/s41598-023-49271-2 (PMC10716428; doi:10.1038/s41598-023-49271-2)
Supplement: Supplementary file 1 — Supplementary Information. [file 41598_2023_49271_MOESM1_ESM.docx]

**Prediction of Chronic Kidney Disease Progression Using Recurrent Neural Network and Electronic Health Records**

**(Supplementary Information)**

Yitan Zhu^1*^, Dehua Bi^2^, Milda Saunders^3^, Yuan Ji^2*^

1. Computing, Environment and Life Sciences, Argonne National Laboratory, Lemont, IL 60439, USA
2. Department of Public Health Sciences, The University of Chicago, Chicago, IL 60637, USA
3. Department of Medicine, The University of Chicago, Chicago, IL 60637, USA

^*^Correspondence: [yitan.zhu@anl.gov](mailto:yitan.zhu@anl.gov), [yji@health.bsd.uchicago.edu](mailto:yji@health.bsd.uchicago.edu)

**Section 1: Transformation of Clinical Variables in EHRs**

Demographics and health behavior variables are categorical. Vital information and lab test variables are numeric*.* All categorical clinical variables are one-hot encoded. A categorical clinical variable with $N$ different levels is represented by $N$ binary indicators. A variable value of the $n$th level is represented by the $n$th indicator being 1 and all other indicators being 0. Some of the numeric variables are log transformed, including blood urea nitrogen, creatinine, ferritin, LDL, cholesterol, parathyroid hormone, phosphorous, triglycerides, uric acid, urine creatinine, urine micro albumin, urine protein, vitamin D25, eGFR, body mass index, and weight. The transformation is $\log_{10} \left( x+1 \right)$, where $x$ is the variable to be transformed. Log transformation makes the variable distributions more symmetric and suppresses the effect of extreme values. For demographics, lab test, and health behavior variables, the extracted EHRs include the date and time when their values were recorded. We transform the date and time into relative time after the patient birth.

**Section 2: Construction of Time Series and Related Data Processing**

Let $t_{\mathrm{lab}\_\min}$ and $t_{\mathrm{lab}\_\max}$ be the earliest and latest times of lab tests. Given a time interval size of $t_{w}$ (e.g. 7 days), the time series is composed of $M$ time intervals, where $M$ can be calculated by

$$M=\left\lceil\frac{t_{\mathrm{lab}\_\max}-t_{\mathrm{lab}\_\min}}{t_{w}} \right\rceil$$

where $\left\lceil\cdot\right\rceil$ is the ceiling function that returns the smallest integer no smaller than the input. The start and end times of the first time interval is $t_{lab\_min}$ and $t_{lab\_min}+t_{w}$, while the start and end times of the last time window is ${t_{lab\_min}+\left( M-1 \right)\times t}_{w}$ and $t_{lab\_min}+M{\times t}_{w}$. We take the middle time of each interval as the time of corresponding time point. The first and last time points are at $t_{\mathrm{lab}\boldsymbol{\_}\min}+0.5\times t_{w}$ and ${t_{lab\_min}+\left( M-0.5 \right)\times t}_{w}$, respectively.

For lab test variables, if multiple readouts of a variable are available during a time interval, their average is calculated to represent the variable in the feature vector. For vital information variables, average values are also calculated if multiple readouts are available in a time interval. Also, if no readout of a vital information variable is available at a time point, a linear interpolation is applied to fill its value based on two non-missing values closest to the time point, one value before the time point in consideration and the other value after the time point if such data are available. Demographics variables, i.e., sex and race, takes the same values in feature vectors across all time points. For health behavior variables, if multiple readouts are available in a time interval, the last readout is used in the feature vector. If no readout is available at a time point, its value was filled by a non-missing value from a previous time point closest to the time point in consideration, if such data are available. After assembling clinical variables and time to form the feature vectors in time series, we remove the time points at which all lab test variables are missing. In the case of only essential variables are used for analysis, the time points where all essential lab test variables are missing are removed.

There are still missing values in the generated time series. For a categorical variable, a missing value is naturally represented by 0 values of all indicators associated with the variable, because the variable does not take any of the levels. For a numerical variable, if missing values exist, they are replaced by the mean value of the variable calculated from the whole patient cohort. A binary indicator is added as a feature to indicate where the variable is missing. After the imputation of missing values, the time series of all variables have 101 features, and the time series of essential variables have 51 features.

**Section 3: Matching Case Patients with Control Patients**

For a case patient, the first time point is required to have an eGFR readout available to indicate the patient disease stage and the transition point must be covered by the prediction window. For a control patient, the first and last time points are required to have an eGFR readout available to indicate the patient disease stage. We match each case patient with four control patients to form the data for predictive analysis. Case patients are ranked based on their series length from long to short and get matched successively in that order. To match a case patient, only control patients who have the same sex and race and whose sequence length is not shorter than that of the case patient (denoted by $L_{\mathrm{case}}$) are considered. Only the latest $L_{\mathrm{case}}$ time points of the candidate control patients are used for matching based on age/time. To do that, we calculate the average time of the time points in the case patient time series and the average times of candidate control patient series. Four of the candidate control patients with average times closest to that of case patient are selected. The latest $L_{\mathrm{case}}$ time points of the selected control patients are used for predictive modeling.

Supplementary Table 1 summarizes the matched data generated using all variables and with $t_{w}=7$ days, $t_{\mathrm{gap}}=7$ days, and $t_{\mathrm{pre}}=365$ days. Supplementary Fig. 1a shows the distribution of the sequence length of time series for case patients. The length of control patient time series follows the same distribution because each case patient is matched with four control patients of the same times series length. Supplementary Fig. 1b shows the distribution of the time duration between the transition point and the last time point in time series for case patients.

**Supplementary Table 1** Summary of matched data generated with all variables, $t_{w}=7$ days, $t_{\mathrm{gap}}=7$ days, and $t_{\mathrm{pre}}=365$ days.

|  | | **Case** | **Control** |
| --- | --- | --- | --- |
| **Time series length (mean and standard deviation)** | | 24.50(19.23) | 24.50(19.23) |
| **Age (mean and standard deviation)** | | 66.38(14.20) | 66.36(14.09) |
| **Number of patients (sex)** | **Male** | 584 | 2,336 |
|  | **Female** | 956 | 3,824 |
| **Number of patients (race)** | **American Indian or Alaska native** | 2 | 8 |
|  | **Asian/Mideast Indian** | 16 | 64 |
|  | **Black/African-American** | 1,157 | 4,628 |
|  | **More than one race** | 11 | 44 |
|  | **White** | 330 | 1,320 |
|  | **Unknown** | 24 | 96 |
| **Total number of patients** | | 1,540 | 6,160 |

Numbers before and in the parentheses are mean and standard deviation, respectively.


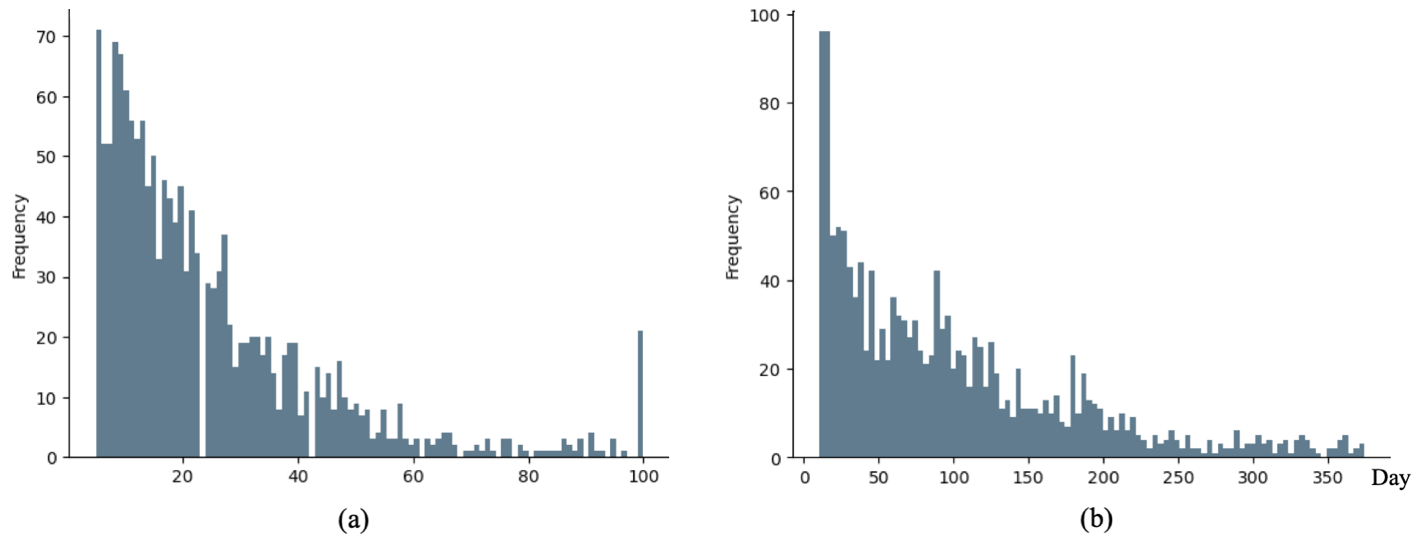


**Supplementary Figure 1** (a) Distribution of the time series length of case patients. (b) Distribution of the time duration between the transition point and the last time point of the time series used in the analysis for case patients.

**Section 4: Generation of Data without Patient Stage IIIb Information**

To generate data without patient stage IIIb information, we basically remove the feature vectors at time points corresponding to stage IIIb from each patient’s time series, so that the remaining feature vectors represent only stage IIIa and earlier stages. To detect stage IIIb time points, we follow the approach of identifying transition to stages IV/V as described in the main text, but use 44 as the cutoff on eGFR instead of 29, to identify the transition point to stages IIIb/IV/V. If a transition to stages IIIb/IV/V is identified, feature vectors of time points after the transition point are excluded from analysis.

Supplementary Table 2 gives the numbers of case and control patients in the matched data generated using different settings.

**Supplementary Table 2** Numbers of patients in the matched data generated with different settings.

| **Variable** | **Stage IIIb data excluded** | **Prediction window (**$\boldsymbol{t}_{\mathbf{pre}}$**)** | **# Case** | **# Control** |
| --- | --- | --- | --- | --- |
| Essential | No | 90 days | 871 | 3,484 |
|  |  | 365 days (1 year) | 1,500 | 6,000 |
|  | Yes | 365 days (1 year) | 191 | 764 |
|  |  | 1,095 days (3 years) | 431 | 1,722 |
|  |  | 1,825 days (5 years) | 554 | 2,201 |
| All | No | 90 days | 946 | 3,784 |
|  |  | 365 days (1 year) | 1,540 | 6,160 |
|  | Yes | 365 days (1 year) | 199 | 796 |
|  |  | 1,095 days (3 years) | 438 | 1,750 |
|  |  | 1,825 days (5 years) | 568 | 2,258 |

**Section 5: Details of Implementing and Training Prediction Models**

Adam optimizer with default parameter setting is used to train LSTM RNN models^1^. The learning rate is initialized at 0.001 and is reduced by a factor of 10 when the reduction of validation loss is smaller than 0.00001 in 10 epochs. The training process stops early if the reduction of validation loss is smaller than 0.00001 in 20 epochs; otherwise, the full training process takes 100 epochs. During model training, patients in a training batch are required to have the same time series length. The predictive modeling pipeline is implemented using the Keras package (<https://keras.io/>) with Tensorflow (<https://www.tensorflow.org/>) backend.

For random forest, we use the Scikit-Learn python package (<https://scikit-learn.org/>) to implement the analysis pipeline. The prediction model includes 200 decision trees and default values are used for all other parameters. For LightGBM, we use the LightGBM python package ([https://LightGBM.readthedocs.io/en/latest/index.html](https://lightgbm.readthedocs.io/en/latest/index.html)) to implement the analysis pipeline. The training of LightGBM model stops early to avoid overfitting if the validation loss does not reduce in 200 boosting steps; otherwise, the whole training process will take 2,000 boosting steps. Default values are used for all other parameters.

**Section 6: Sequential Forward Variable Selection Procedure**

The sequential forward variable selection is performed via a two-tier cross-validation analysis. For each out-tier cross-validation trial, a whole sequential forward variable selection procedure is performed. The procedure starts by identifying the most predictive single variable, and then additional variables are added one by one until 20 variables are selected. At each step of the procedure, the inner-tier cross-validation takes an exhaustive search to identify a variable, the addition of which generates the highest prediction performance. A model is then trained with the identified variable added, and its prediction performance is evaluated using the out-tier testing set. For health behavior variables, only the main variables indicating the usage status of drug, alcohol, and smoking are considered for variable selection, while variables indicating the types of drugs and smoking are not considered for variable selection.

The RNN architecture used to train prediction models in the variable selection process follows the framework shown in Fig. 3b, but the numbers of units and nodes in the layers commensurate with the number of input features. The number of units/nodes in the LSTM layer and the first dense layer is equal to the number of input features. The numbers of nodes in the second and third dense layers are $\left\lfloor K-(K-2)/3 \right\rfloor$ and $\left\lfloor K-2\times\left( K-2 \right)/3 \right\rfloor$, respectively, where *K* is the number of input features and $\left\lfloor\cdot\right\rfloor$ is the floor function that returns the largest integer no larger than its input. The parameter setting used for training the RNN models is the same as shown in Section 5 of the Supplementary Information.

Supplementary Table 3 shows the frequencies of the variables being selected across cross-validation trials in the sequential forward search process.

**Supplementary Table 3** Most frequently selected variables in the sequential forward search process.

| **Number of Selected Variables** | **Most Frequently Selected Variables** |
| --- | --- |
| 1 | eGFR(100%) |
| 2 | eGFR(100%), Smoking status(26.67%), … |
| 3 | eGFR(100%), Smoking status(36.67%), Calcium(23.33%), … |
| 4 | eGFR(100%), Smoking status(46.67%), Calcium(30%), Alcohol usage status(23.33%), … |
| 5 | eGFR(100%), Smoking status(60%), Calcium(36.67%), Alcohol usage status(26.67%), Systolic blood pressure(23.33%), … |
| 6 | eGFR(100%), Smoking status(63.33%), Calcium(40%), Systolic blood pressure(30%), Alcohol usage status(26.67%), Hgb(23.33%), … |
| 7 | eGFR(100%), Smoking status(63.33%), Calcium(43.33%), Systolic blood pressure(40%), Alcohol usage status(33.33%), Blood urea nitrogen(26.67%), Hgb(26.67%), … |
| 8 | eGFR(100%), Smoking status(66.67%), Calcium(43.33%), Systolic blood pressure(40%), Alcohol usage status(33.33%), Blood urea nitrogen(33.33%), Hgb(33.33%), Urine creatinine(30%), … |
| 9 | eGFR(100%), Smoking status(70%), Systolic blood pressure(46.67%), Calcium(43.33%), Alcohol usage status(40%), Hgb(36.67%), Urine creatinine(36.67%), Blood urea nitrogen(33.33%), Creatinine(30%), … |
| 10 | eGFR(100%), Smoking status(70%), Systolic blood pressure(50%), Alcohol usage status(46.67%), Calcium(46.67%), Blood urea nitrogen(36.67%), Creatinine(36.67%), Hgb(36.67%), Urine creatinine(36.67%), Urine micro albumin(36.67%), … |
| 11 | eGFR(100%), Smoking status(73.33%), Systolic blood pressure(50%), Calcium(50%), Alcohol usage status(46.67%), Creatinine(43.33%), Blood urea nitrogen(40%), Urine creatinine(40%), Hgb(36.67%), Urine micro albumin(36.67%), Albumin(33.33%), … |
| 12 | eGFR(100%), Smoking status(76.67%), Systolic blood pressure(56.67%), Calcium(53.33%), Alcohol usage status(50%), Creatinine(46.67%), Blood urea nitrogen(43.33%), Urine creatinine(43.33%), Albumin(40%), RACE(40%), Urine micro albumin(40%), Hgb(36.67%), … |
| 13 | eGFR(100%), Smoking status(76.67%), Systolic blood pressure(63.33%), Urine creatinine(56.67%), Calcium(53.33%), Alcohol usage status(50%), Blood urea nitrogen(50%), Creatinine(46.67%), Race(46.67%), Urine micro albumin(46.67%), Albumin(43.33%), Saturation(43.33%), Uric acid(40%), … |
| 14 | eGFR(100%), Smoking status(76.67%), Systolic blood pressure(66.67%), Calcium(60%), Urine creatinine(60%), Urine micro albumin(53.33%), Alcohol usage status(50%), Blood urea nitrogen(50%), Race (50%), Albumin(46.67%), Creatinine(46.67%), Saturation(46.67%), Uric acid(43.33%), Hgb(40%), … |
| 15 | eGFR(100%), Smoking status(80%), Systolic blood pressure(70%), Calcium(66.67%), Urine creatinine(66.67%), Alcohol usage status(53.33%), Creatinine(53.33%), Race(53.33%), Urine micro albumin(53.33%), Blood urea nitrogen(50%), Uric acid(50%), Albumin(46.67%), Saturation(46.67%), Body mass index(43.33%), Hgb(43.33%), … |
| 16 | eGFR(100%), Smoking status(83.33%), Calcium(73.33%), Systolic blood pressure(70%), Urine creatinine(66.67%), Race(63.33%), Alcohol usage status(53.33%), Albumin(53.33%), Creatinine(53.33%), Uric acid(53.33%), Urine micro albumin(53.33%), Blood urea nitrogen(50%), Ferritin(50%), LDL(50%), Saturation(50%), Body mass index(46.67%), … |
| 17 | eGFR(100%), Smoking status(86.67%), Calcium(73.33%), Urine creatinine(73.33%), Systolic blood pressure(70%), Race(63.33%), Creatinine(60%), Alcohol usage status(56.67%), Albumin(56.67%), Uric acid(56.67%), Bicarbs CO2(53.33%), Blood urea nitrogen(53.33%), LDL(53.33%), Urine micro albumin(53.33%), Ferritin(50%), Hgb(50%), Saturation(50%), … |
| 18 | eGFR(100%), Smoking status(86.67%), Urine creatinine(76.67%), Systolic blood pressure(73.33%), Calcium(73.33%), Albumin(66.67%), Creatinine(66.67%), Race(63.33%), Uric acid(60%), Alcohol usage status(56.67%), LDL(56.67%), Urine micro albumin(56.67%), Body mass index(53.33%), Bicarbs CO2(53.33%), Blood urea nitrogen(53.33%), Ferritin(53.33%), Oxygen saturation(50%), Hgb(50%), … |
| 19 | eGFR(100%), Smoking status(86.67%), Urine creatinine(76.67%), Systolic blood pressure(73.33%), Calcium(73.33%), Creatinine(73.33%), Albumin(70%), Race (70%), Alcohol usage status(60%), Bicarbs CO2(60%), Uric acid(60%), Urine micro albumin(60%), LDL(56.67%), Triglycerides(56.67%), Vitamin D25(56.67%), Body mass index(53.33%), Blood urea nitrogen(53.33%), Ferritin(53.33%), HDL(53.33%), … |
| 20 | eGFR(100%), Smoking status(90%), Calcium(76.67%), Creatinine(76.67%), Urine creatinine(76.67%), Albumin(73.33%), Systolic blood pressure(73.33%), Race(70%), Uric acid(70%), Alcohol usage status(63.33%), Bicarbs CO2(63.33%), LDL(63.33%), Urine micro albumin(60%), Urine RBC count(60%), Vitamin D25(60%), Body mass index(56.67%), Blood urea nitrogen(56.67%), Ferritin(56.67%), HDL(56.67%), HbgA1C(56.67%), … |

Percentages in parentheses indicate the frequencies of the variables being selected across cross-validation trials. Variables are ordered by the selection frequency.

**References**

1 Ba, J. & Kingma, D. in *International Conference on Learning Representations (ICLR)* (2015).
